# Photoluminescence of a Uranium(IV) Alkoxide Complex

**DOI:** 10.1021/jacsau.4c01022

**Published:** 2024-12-12

**Authors:** Leyla R. Valerio, Sabyasachi Roy Chowdhury, Rob Lewis, Kathryn E. Knowles, Bess Vlaisavljevich, Ellen M. Matson

**Affiliations:** † Department of Chemistry, University of Rochester, Rochester, New York 14627, United States; ‡ Department of Chemistry, University of South Dakota, Vermillion, South Dakota 57069, United States; § Department of Chemistry, University of Iowa, Iowa City, Iowa 52240, United States

**Keywords:** actinides, uranium, photoluminescence, f–f excitation, CASSCF/CASPT2 calculations

## Abstract

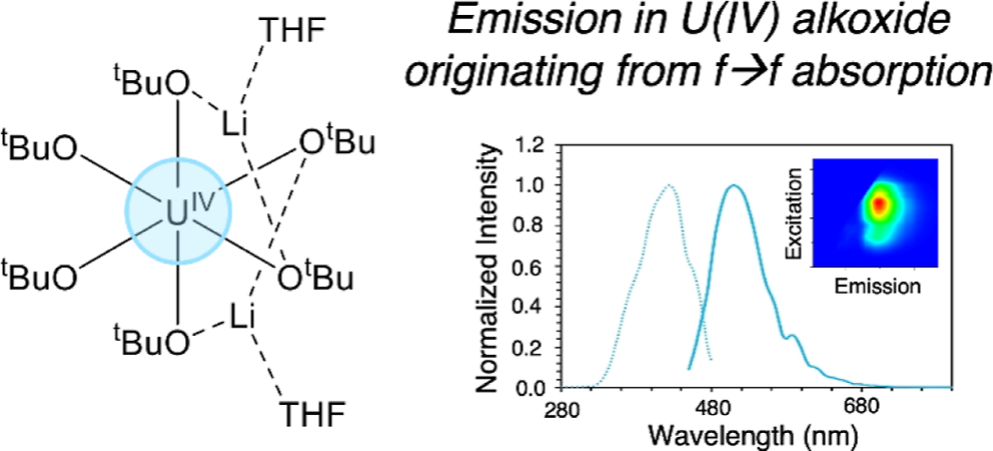

In this report, we describe the photoluminescence of a homoleptic uranium(IV) alkoxide complex. Excitation of [Li(THF)]_2_[U^IV^(O^*t*^Bu)_6_] leads to the first example of photoluminescence from a well-defined actinide complex originating from an f–f excitation, supported by second order multiconfigurational electronic structure calculations including spin–orbit coupling. These calculations show strong spin–orbit coupling between the excited triplet and singlet states for the 5f-orbital manifold, which leads to a long-lived excited state lifetime of 0.85 s at low temperature. The photophysical properties of homoleptic uranium(V) and uranium(VI) tertbutoxide complexes are also presented; we find that oxidation of the uranium(IV) alkoxide results in quenching of luminescence in [Li(THF)][U^V^(O^*t*^Bu)_6_] and [U^VI^(O^*t*^Bu)_6_]. This is attributed to competing ligand to metal charge transfer absorption processes shifted to lower energy upon oxidation of the actinide center, which mask the relevant f–f transitions in the visible region of the electronic absorption spectrum.

## Introduction

Recent efforts in actinide chemistry have focused on studying the electronic structure of metal–ligand complexes and have proven to be useful in elucidating their fundamental spectroscopic properties in a wide range of oxidation states.^1−10^ To this point, the electronic absorption spectra of a diverse range of uranium complexes have been well reported in the literature.^11−17^ The electronic transitions within these complexes result from three main types of absorption mechanisms: intraconfigurational f–f transitions, interconfigurational f-d transitions, or charge transfer transitions arising from actinide-ligand orbital mixing (Figure S1).^18^ Both f-d and charge transfer transitions are orbitally allowed, resulting in significantly higher molar extinction coefficients compared to Laporte forbidden f–f transitions. It has been observed, however, that the ligand environment has an impact on the intensity of f–f bands in actinide complexes, and that ligand absorption can readily compete with f–f absorption if sufficient overlap of the transitions occurs.^19^

Although the area remains significantly underdeveloped, the actinide community has a growing interest in the photophysical properties of uranium complexes to couple with the understanding of their absorption spectra. The lack of reports in this area stands in contrast to the vast body of work that has been devoted to the photophysical properties of transition-metal and lanthanide complexes for a broad range of applications.^20−32^ Moreover, fundamental electronic structure information is obtained, that through synergistic comparisons with theory can inform future synthetic efforts and modeling choices (e.g., quantum chemical method, solvent model, etc.). To this point, a special emphasis has been placed on luminescent lanthanide complexes historically due to their distinct spectroscopic properties. Absorption and emission bands that correspond to Laporte forbidden f–f transitions, large Stokes’ shifts, and ligand-sensitized emission are all unique phenomena characteristic of many emissive lanthanide complexes.^33−36^ Understanding f–f absorption and the mechanisms of relaxation from excited states is crucial for elucidating electronic structure and photophysical behaviors in uranium complexes, particularly as chemists seek to exploit these properties in potential applications across various fields such as photocatalysis and optoelectronics, akin to the lanthanide elements.

U(VI) (5f^0^) compounds, namely the aqueous uranyl(VI) dication, represent the most well-studied photoluminescent actinides, with emission arising from ligand-to-metal charge transfer (LMCT) transitions.^37−40^ Comparison of the electronic spectra of a wide variety of uranyl complexes shows characteristic features, with absorption and emission spectra that occur between 350-500 nm and 470–650 nm, respectively.^41^ The uranyl moiety is highly sensitive to its coordination environment, temperature, and solvent. For example, the nonluminescent nature of aqueous uranyl(VI) complexes with carbonate or chloride ligands is well-known, with quenching occurring as a result of rapid intermolecular electron transfer.^42,43^ Comparatively, studies detailing emission of the uranyl(VI) ion in nonaqueous media are less common, as solvent has been shown to play a role in hindering emission to different extents. For example, Palit and co-workers detail the emission of UO_2_(NO_3_)_2_ in methanol and observe broadened emission bands due to stronger interactions between the solvent molecules and the uranyl ion (solvent-uranyl charge transfer). Upon excitation, they also observe degradation of their sample due to photooxidation of methanol by the excited state of the uranyl ion, (UO_2_^2+^)*.

Reports of luminescent actinide(IV) (5f^2^) compounds are exceedingly rare in comparison to those focused on the uranyl(VI) ion. For example, Schelter and co-workers have described the photophysics of luminescent Th(IV) (5f^0^) anilido and imido complexes, assigning the origin of emission to intraligand *n* → π*.^44^ More recently, our group described photoluminescent pyridine dipyrrolide Th(IV) complexes, whose emission results from intraligand and ligand-to-ligand charge transfer events.^17^ It is important to note that in all reported examples of Th(IV) luminescence, emission results from excitation localized on the organic ligands, as the metal ion itself does not contribute to emissive low-energy excited states. Interestingly, only a handful of studies have characterized luminescence in U(IV) compounds, with all reports involving some degree of charge transfer in the absorption process which leads to emission. For example, a representative work by Hashem et al. details the electronic absorption and emission of a series of uranium(IV) halide complexes in nonaqueous media.^45^ Individual electronic transitions were assigned via second order multireference methods (CASPT2). The decay of excited states with charge transfer and d-orbital character into the 5f^2^-manifold results in the observed UV–visible emission spectra. Subsequent bonding analysis based on density functional theory (DFT) included natural bond orbital (NBO) analysis and Bader’s quantum theory of atoms in molecules (QTAIM), supporting a dative bond between U(IV) and chloride, albeit with some degree of orbital mixing.^45^ On the other hand, Kirishima et al. reported the first comprehensive photoluminescence study of aqueous U(IV) ions where the luminescence resulted from metal-centered f–f excitations.^46^ Specifically, when the excitation is performed to populate the ^1^S_0_ state from the ^3^H_4_ state (at 245 nm), emission is observed into the 5f-manifold. Emission from reduced [U^V^O_2_]^+^ (5f^1^) complexes is also known.^47,48^

Given the striking differences in photophysical properties of U(IV) halide and aquo complexes, we opted to examine an intermediate case with anionic oxygen-based ligands, hoping that they could contribute to our understanding given the scarcity of photophysical measurements. Alkoxide ligands have found widespread use in actinide science, making up some of the earliest synthetic targets in nonaqueous actinide chemistry due to their ability to stabilize a wide variety of oxidation states of uranium.^49−52^ Herein, we expand upon prior synthetic work by reporting the photophysical characterization of a homoleptic uranium(IV) alkoxide complex. Emission from the alkoxide complex results from a metal-centered f–f excitation in nonaqueous solvent, making this the first example that is not coupled with charge transfer contributions. Moreover, investigation of the uranium alkoxide complex at low temperatures demonstrates photoluminescence with an unprecedented long-lived excited state, which we credit to spin–orbit coupling leading to significant triplet-singlet mixing in the excited state 5f-orbital manifold. U(V) and U(VI) congeners of the uranium alkoxide were also studied but no luminescence was observed, indicating an oxidation state dependency of the emissive behavior for the U(IV) complex. Collectively, this work reveals novel structure–function relationships that provide insight into synthetic handles that control the emission in uranium complexes, which may prove useful in the design of actinide-based emitters.

## Experimental Section

### Safety Considerations

Caution! Depleted uranium (primary isotope ^238^U) is a weak α-emitter (4.197 MeV) with a half-life of 4.47 × 10^9^ years; manipulations and reactions should be carried out in monitored fume hoods or in an inert atmosphere drybox in a radiation laboratory equipped with α- and β counting equipment.

### General Considerations

All air- and moisture-sensitive manipulations were carried out using standard high vacuum line, or Schlenk techniques or in an MBraun inert atmosphere drybox containing an atmosphere of purified dinitrogen. Solvents for air- and moisture-sensitive manipulations were dried and deoxygenated using a glass contour solvent purification system (Pure Process Technology, LLC) and stored over activated 4 Å molecular sieves (Fisher Scientific) prior to use. Deuterated solvents for ^1^H NMR spectroscopy were purchased from Cambridge Isotope Laboratories and stored in the glovebox over activated 3 Å molecular sieves after three freeze–pump–thaw cycles. Chemicals were purchased from commercial sources and solids were dried under high vacuum before being brought into the glovebox. LiO^*t*^Bu was prepared by deprotonation of HO^*t*^Bu (Sigma-Aldrich) with *n*-butyllithium solution (2.6 M in hexanes). UCl_4_, [Li(THF)]_2_[U^IV^(O^*t*^Bu)_6_], [Li(THF)][U^V^(O^*t*^Bu)_6_], and [U^VI^(O^*t*^Bu)_6_] were synthesized following reported procedures.^52,53^

### Physical Measurements

^1^H NMR spectra were recorded at room temperature on a 400 MHz Bruker AVANCE spectrometer or a 500 MHz Bruker AVANCE spectrometer locked on the signal of deuterated solvents. All the chemical shifts are reported relative to the chosen deuterated solvent as a standard. Electronic absorption measurements were recorded at room temperature in anhydrous tetrahydrofuran in sealed 1 cm quartz cuvettes using an Agilent Cary 6000i UV–vis–NIR Spectrophotometer. Emission spectra were collected in 1 cm quartz cuvettes with a Spex Fluoromax-3 fluorometer (Horiba) with a photomultiplier tube detector. Time-resolved emission measurements were acquired using two different home-built optical setups. Time-correlated single-photon counting measurements were used to measure emission on time scales up to 100 ns. Samples for TCSPC were dissolved in anhydrous tetrahydrofuran solution and placed in 1 cm quartz cuvettes, and photoexcited by a defocused laser beam provided by a pulsed laser diode (PicoHarp 300, PDL 800-D, 405 nm) to a photomultiplier tube detector. Samples for longer time scale measurements (up to 1 s) were dissolved in anhydrous 2-MeTHF solution and placed in J Young tubes. A 65 v5 A DC power supply and GCA/McPherson instrument photomultiplier housing (model EU-701-53) were used. A 1544 Strobotac flashlamp was used as the light source. Data was collected on a Tektronix TBS 1102B-Edu (100 MHz, 2 GS/s) digital oscilloscope.

## Computational

### Density Functional Theory (DFT)

Gas phase geometry optimizations were performed for the ground state triplet with the PBE0-D3 functional.^54,55^ The resolution of the identity (RI) approximation was used for integral evaluation.^56^ The def2-TZVP basis set was used for all atoms with the exception of uranium and hydrogen atoms. While the def-TZVP basis set was used for uranium,^57,58^ the hydrogen atoms were described by def2-SV(P) basis sets.^59^ The corresponding effective core potentials (ECPs) are employed to recover scalar relativistic effects.^59^ The SCF energy was converged to 10^–7^ a.u., while the Cartesian gradient was converged to 10^–4^ a.u. All structures were confirmed as minima by harmonic vibrational analysis. DFT calculations were performed as implemented in the Turbomole program package.^60^

### Multiconfigurational Methods

To interpret the measured optical spectra, computations using multireference wave function-based methods were performed. First, state-average complete active space self-consistent field (SA-CASSCF) calculations were performed on the DFT-optimized geometry.^61^ The active space included the seven 5f orbitals and corresponding two electrons, denoted (2e,7o). SA-CASSCF computations allows for the computation of 21 triplet and 28 singlet scalar-relativistic states arising from f–f excitations. To account for dynamic electron correlation, the SA-CASSCF energy levels were subjected to extended multistate second-order perturbation theory (XMS-CASPT2) calculations.^61−63^ In the XMS-CASPT2 calculations, the zero-order Hamiltonian included an IPEA shift of 0.25 and an imaginary shift of 0.2 au^64,65^ Scalar-relativistic effects were included by employing Douglas–Kroll–Hess (DKH) Hamiltonian together with the relativistic ANO-RCC basis sets.^66^ Specifically, the following contractions were used: 9s8p6d4f2g1h for U, 4s3p2d1f for O and Li, 2s1p for C, and 1s for H atoms. Cholesky decomposition along with local exchange screening were employed to reduce the cost of integral evaluation.^67^ All wave function-based computations were performed as implemented in the OpenMolcas program package.^68^

### Spectral Modeling

The scalar-relativistic states obtained via XMS-CASPT2 were then utilized to obtain the spin–orbit (SO) mixing of the states a posteriori via a state-interaction Hamiltonian.^69^ The diagonal elements of the effective Hamiltonian are replaced with the XMS-CASPT2 energies, following the so-called SO-XMS-CASPT2 approach.^62,63,69,70^ Additionally, the transition dipole moments, and oscillator strengths between SO-states were computed, allowing for the prediction of the absorption and emission spectrum. Specifically, the absorption spectra is predicted by computing the allowed excitations from the SO-XMS-CASPT2 ground state (consisting of contributions from the lowest spin-free triplet and singlet states). Traditionally, one performs a geometry optimization on the excited state populated prior to computing an emission spectrum; however, the excited states obtained herein are all f–f transitions and the spin-free states are coupled. Therefore, we assume structural relaxation in the excited state is negligible and compute the emission spectrum directly. Therefore, the transition dipole moments corresponding to emission are computed from the state giving the highest intensity in the absorption spectrum at 486 nm.

## Results and Discussion

### Photophysical Characterization of [Li(THF)]_2_[U(O^*t*^Bu)_6_] (**1**)

**Figure 1:**
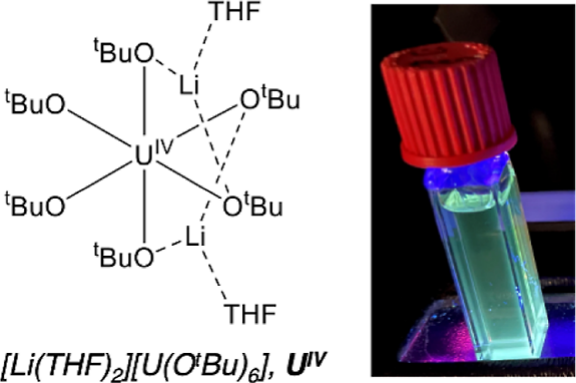
[Li(THF)]_2_[U(O^*t*^Bu)_6_] under irradiation with 365 nm light.

Our interest in the photophysical properties of uranium(IV) alkoxide complexes came from a fortuitous discovery; our group had synthesized the lithium salt of the uranium(IV) tertbutoxide anion, [Li(THF)]_2_[U^IV^(O^*t*^Bu)_6_] (**1**), as a starting material for a separate project (Figure 1). Interestingly, analysis of a sample of **1** in tetrahydrofuran under ultraviolet irradiation revealed emission from this complex.

**Figure 2:**
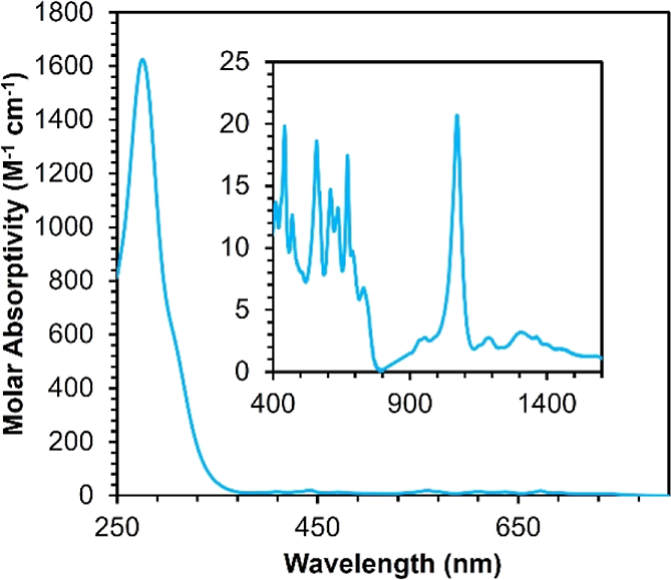
Electronic absorption spectrum of **1** collected at room temperature in anhydrous THF. Inset shows weak f–f transitions resulting from the 5f^2^ electronic configuration of the uranium metal center in the visible and near-infrared regions.

The absorption spectrum of **1** was not disclosed by Hayton and co-workers in their initial report.^52^ Due to the importance of this data for understanding the excited state electronic structure and emission behavior of molecular inorganic complexes, we evaluated the electronic absorption spectrum of **1** in tetrahydrofuran (THF, Figure 2). At low concentrations (O.D. < 1.0), **1** exhibits a single broad absorption centered at 275 nm (ε = 1628 M^–1^ cm^–1^). An additional feature is present at 305 nm (ε = 595 M^–1^ cm^–1^) in the spectrum, appearing as a shoulder of the main absorption band. The absorption profile of **1** resembles that described for other nonaqueous six-coordinate uranium(IV) complexes, including [Li(THF)_4_][UCl_5_(THF)] (λ_max_ = 277 and 303 nm) and [UCl_6_]^2–^ (λ_max_ = 280 and 307 nm).^45^ In these examples, the authors assign the observed high-energy absorption feature as an f-d transition originating from a ^3^H_4_ ground state to a ^3^F_2_ Russell–Saunders coupled state, with the low-energy transition designated as a halide to uranium charge transfer process. The hypsochromic shift of absorption features in **1** is attributed to the increased electronegativity and π-donating properties of the *tert*-butoxide ligands in comparison to the chloride ligands of [UCl_5_(THF)]^1–^ and [UCl_6_]^2–^.

When the electronic absorption spectrum of **1** is collected at higher concentrations, a range of bands assigned to intraconfigurational f–f transitions resulting from the 5f^2^ electronic configuration of the uranium metal center are observed (Figure 2, inset). The f–f transitions of **1** display a strikingly similar pattern to those reported for [UCl_5_(THF)]^1–^,^45^ showing that the ligands surrounding the metal center have a negligible effect on the energies of the weaker f–f electronic transitions within each complex. The minor perturbations in these transitions demonstrates the ionic nature of the bonds between the U(IV) metal center and coordinated ligands. The molar absorptivities of these transitions, however, are quite different. Extinction coefficients reported for [UCl_5_(THF)]^1–^ range from 1 to 8 M^–1^ cm^–1^ whereas these values range from 10 to 23 M^–1^ cm^–1^ for **1**, suggesting that these intraconfigurational transitions in the uranium(IV) tertbutoxide complex are more “allowed”.

**Figure 3:**
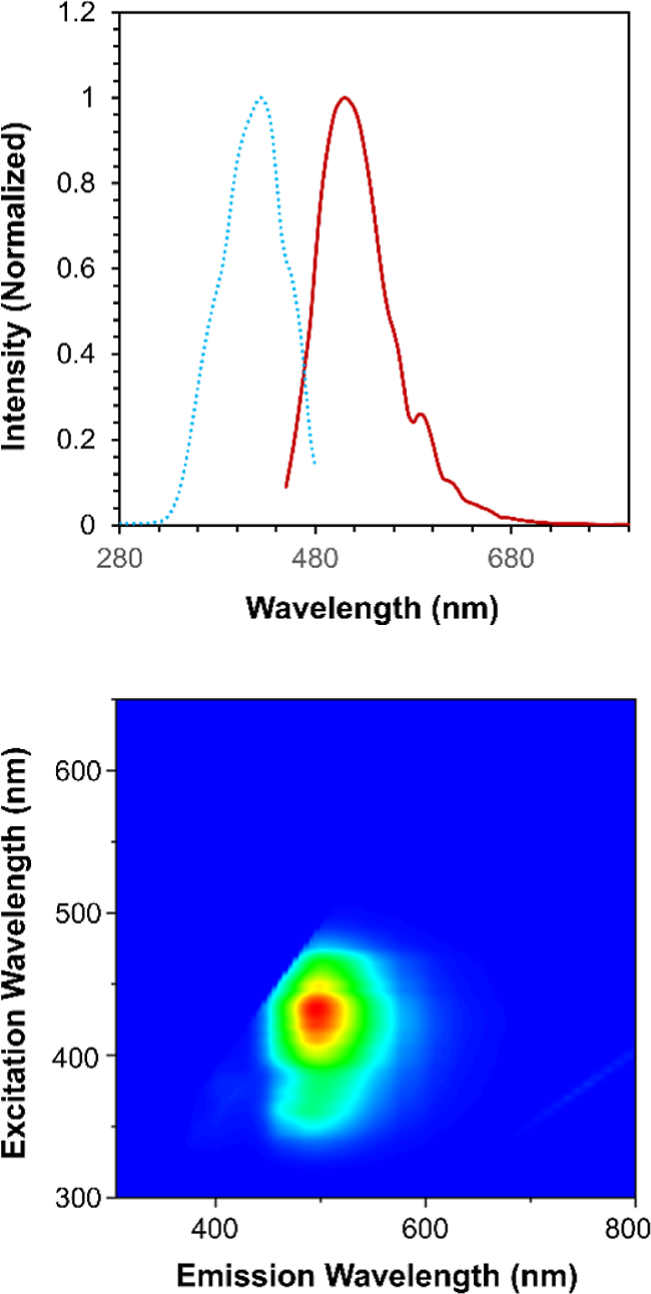
Emission (solid red line) and excitation (dashed blue line) spectrum of **1** recorded in anhydrous THF at room temperature (top). Photoluminescence emission map measured in anhydrous THF at room temperature. Red represents excitation wavelengths that result in peak emission intensity (bottom).

With an understanding of the electronic absorption spectrum of **1** in hand, we returned to our initial aim of understanding the photoluminescence of the uranium(IV) alkoxide complex. To our surprise, excitation of **1** into the ligand-to-metal charge transfer band located at 280 nm at room temperature resulted in no appreciable emission. This result contrasts with the behavior of uranium(IV) chloride complexes reported by Hashem et al. for [UCl_5_(THF)]^1–^ (λ_exc_ = 260–350) nm, where emission results from excitation into the Cl → U charge transfer bands located in the ultraviolet region.^45^ Instead, when the solution of **1** is excited at lower energies (λ_exc_ = 350–480 nm), strong photoluminescence is observed (Figure 3). The emission spectrum possesses one major feature centered at 500 nm, with a series of shoulders appearing at 550, 590, and 620 nm. The excitation spectrum at 500 nm reveals that emission results from an absorption at 425 nm, correlating to the f–f electronic transition in the electronic absorption spectrum of **1** (vide supra). To determine whether the shoulders in the emission spectrum originate from multiple independent excited states or result from vibrational progressions, excitation spectra were recorded monitoring emission at each of the bands (Figure S3). These data reveal that all emission peaks correspond to a single excited state, suggesting that the overlapping features are of the same orbital parentage. A photoluminescence emission (PLE) map of **1** was also collected, confirming that excitation of the sample at 425 nm results in an emission event at 500 nm, with no other observable emission resulting from this excitation (Figure 3).

**Figure 4:**
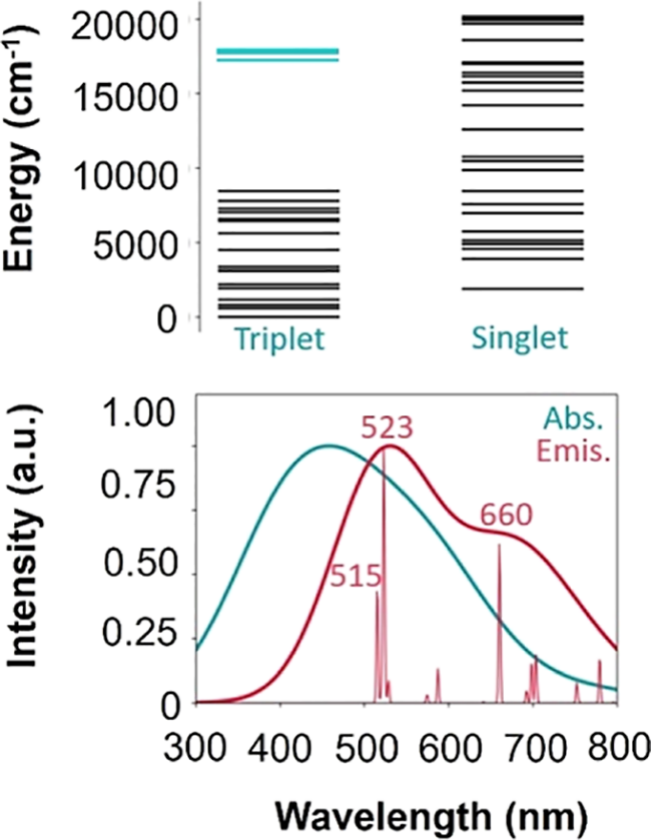
(top) Triplet and singlet f-manifolds; triplet states contributing to the calculated maximum absorption (λ_calc_) are highlighted in teal, (bottom) calculated SO-XMS-CASPT2 absorption (teal) and emission spectra (red). Note that calculated emission spectrum is from the calculated spin–orbit state (λ_calc_). A broadening of 60 eV is used for absorption and emission with the intensity taken as the normalized oscillator strengths. The emission spectrum is plotted a second time with a 1 eV broadening to show individual transitions. The three individual transitions with largest intensities are labeled.

To gain insight into the origin of the observed electronic transitions in the electronic absorbance spectrum of **1**, UV–vis spectra were computed at the XMS-CASPT2 level of theory. The molecular geometry of the triplet ground state was optimized with DFT for [Li_2_(THF)_2_][U^IV^(O^*t*^Bu)_6_], which is in excellent agreement with the solid–state characterization of **1**. For example, the average U–O bond distances are 2.262 Å with DFT, only 0.001 Å from experiment. We note that good geometries were obtained regardless of whether Li ions and THF molecules were included explicitly (deviation of the average U–O distance by 0.01 Å). On this structure, the SO-XMS-CASPT2 absorption spectrum was computed with the calculated maximum absorption (λ_calc_) appearing at 486 nm (Figure 4 bottom, teal). This is intriguing given that the dominant absorptive transition observed experimentally in the excitation spectrum of **1** appears at 425 nm. Importantly, this calculated value is within expected deviation from what is observed experimentally. The corresponding spin–orbit state that is populated can be assigned as a ^3^P state consisting of 76% triplet and 11% singlet character. Specifically, the triplet contributions consist of three spin-free states at 585, 568, and 561 nm (17,084; 17,615; and 17,833 cm^–1^) (the three states highlighted in Figure 4 top), which are destabilized by mixing with higher energy singlet states leading to the spin–orbit state at 486 nm. This supports an assignment of an f–f transition as the dominating absorptive transition in the experimental spectrum of **1**. Collectively, the experimental results indicate that the emission observed in **1** is a result of the direct excitation of an f-electron within the 5f-orbital manifold. This can also be directly confirmed using theory. The calculated emission spectrum (Figure 4 bottom, red) reproduces the experimentally observed features with peaks appearing at 515, 523, and 660 nm. All of the peaks involve a decay within the f-manifold and involve no charge transfer character. Since transitions were only computed from the spin–orbit λ_calc_ state at 486 nm, this confirms that emission arises purely from f–f transitions.

Complex **1** exhibits a Stokes’ shift of 3528 cm^–1^ (75 nm), which is smaller than the values reported for both [UCl_5_(THF)]^1–^ (Stokes’ shift = 5606 cm^–1^, 62 nm) and [UCl_6_]^2–^ (Stokes’ shift = 5530 cm^–1^, 61 nm).^45^ This difference suggests that **1** undergoes less structural rearrangement following the excitation of an f-electron compared to the excited states of [UCl_5_(THF)]^1–^ and [UCl_6_]^2–^. The uranium(IV) halide complexes are expected to undergo larger structural changes prior to emission involving ligand to metal charge transfer processes. However, Stokes’ shifts ranging from 40 to 100 nm (2,500–10,000 cm^–1^) are typical for reports of the photophysical properties of actinide complexes in the literature.^44,45^ Note that the XMS-CASPT2 calculated Stokes shift for **1** was 37 nm despite allowing no structural relaxation at all (Figure 4, bottom).

The emissive metal-centered excitation observed in complex **1** is a unique finding, as the majority of photoluminescent uranium complexes result from ligand to metal charge transfer absorption processes. Photoluminescence of high-valent uranium complexes, namely [UO_2_]^2+^ (5f^0^) ions, are well established, with the emission spectrum resulting from the decay of the excited state triplet that is formed following a ligand to metal charge transfer event [O_yl_ → U(5f)].^47,48^ Even in reduced states, where 5f electron density is present rendering f–f excitations accessible, ligand to metal charge transfer dominates the majority of transitions for these reduced complexes. For example, the photoluminescence of a pentavalent uranyl amide complex, [K(2.2.2-cryptand)]_2_[UO_2_(N{SiMe_3_}_2_)_3_], was recently reported by Ortu et al.; it was determined that LMCT events govern the absorption properties of the compound and the excited states of the complex are a result of LMCT transitions (amide → U(5f) and O_yl_ → U(5f)).^48^ Similar LMCT-derived mechanisms of excitation leading to photoactive complexes are reported for both [UCl_5_(THF)]^1–^ and [UCl_6_].^2^

There is only one prior example that describes emission from a tetravalent uranium complex resulting from an f–f absorption.^46^ Kirishima et al. detail the in situ generation of U^IV^ ions in aqueous perchlorate solution via hydrogen reduction of [UO_2_]^2+^; excitation of an f-electron in U^IV^ from the ^3^H_4_ ground state to the ^1^S_0_ state with 245 nm light results in ten broad emission features, ranging from 290 to 525 nm. Importantly, the authors note that there was no observable emission when other excitation wavelengths were employed. Notably, the band shapes between the in situ generated U^IV^ ions and **1** are similar; broadened emission bands contrast with the sharp and narrow features that are observed for f–f transitions in lanthanide complexes. This observed difference could be due to the more significant impact that the local coordination environment plays within actinide metals compared to lanthanides.

In THF at room temperature, the quantum yield of **1** is 10% (Φ_PL_ = 0.10; Figure S4). The quantum efficiency of **1** is quite high, particularly when considering the nature of the electronic transition responsible for photoluminescence. By comparison, the quantum yields for both [UCl_5_(THF)]^1–^ and [UCl_6_]^2–^ were unable to be measured due to the weakly emissive properties of both complexes. Historically, metal-centered excited states in tetravalent uranium complexes have been shown to proceed through nonradiative decay pathways involving the 5f-orbital manifold, causing rapid deactivation to the ground state and quenching of luminescence.^71^ The measurable quantum yield for **1** indicates that this compound undergoes different excited state dynamics and nonradiative decay pathways compared to previous reports of tetravalent uranium complexes.

### Lifetime Measurements

To better understand this excited state decay mechanism, time-correlated single-photon counting (TCSPC) experiments were performed. A biexponential decay is observed, with a lifetime of τ_1_ = 715 ps and a second lifetime of τ_2_ = 6.72 ns (Figure S5). These measured lifetimes are consistent with [UCl_5_(THF)]^1–^ and [UCl_6_]^2–^ (τ = 2–10 ns) and aqueous U(IV) ions (τ < 20 ns). Hashem et al. briefly comment on these measurements in their work; the authors suggest the possibility of multiple radiative processes occurring in fluid solution and that radiative decay could involve more than one excited state as a result of emission bands that encompass a myriad of excited state configurations or aggregate emission.^45^ Accordingly, we set out to unravel the complicated solution-state dynamics that could be potentially influencing emission in **1** and its related complexes.

The biexponential decay of **1** may result from the presence of two distinct emissive cmoplexes in solution at room temperature that are close in energy and are in rapid equilibrium at the nanosecond time scale. A similar decay mechanism has been proposed in the case of a luminescent U^IV^ complex, [U(DO_3_A)]Br (DO_3_A = [4,7,10-tris-carboxymethyl-,1,4,7,10-tetraaza-cyclododec-1-yl]-acetic acid).^72^ The authors propose that the observed biexponential decay is due to the existence of two coordination isomers of the U^IV^ species in solution that are in exchange at the nanosecond time scale at room temperature, however the specific isomers were not discussed. They also hypothesized that the emissive minor component could stem from allowed LMCT transitions from the carboxylate ligands to the uranium metal center and the emissive major component as metal-based in origin, evidenced by the longer observed lifetime at room temperature (12 ns). To address the aforementioned possibilities for **1**, variable-temperature ^1^H NMR spectroscopy (25 to −80 °C) was performed to determine whether a second species might be present in solution. As the temperature was decreased, no additional signals were observed (Figure S6), consistent with only a single isomer of **1** in solution.

In their original report describing the synthesis of **1**, Hayton and co-workers discuss that the lithium cations of **1** are either completely solvated or are contained within the secondary coordination sphere and rapidly exchanging between the binding sites formed by the alkoxide oxygen ligands, evidenced by the single peak they observe in both the ^1^H NMR and ^7^Li NMR spectra.^52^ We postulated that this exchange behavior could potentially be the origin of the multiple excited states that we observe. To investigate the importance of the concentration of lithium cations in solution, we performed ^1^H NMR and photoluminescence spectroscopies of **1** in the presence of excess amounts of LiPF_6_ (10 equiv wrt **1**; Figure S7). In both cases, we observe no significant differences in the NMR and emission spectra, consistent with only one species in solution. To investigate the effect that sequestering the coordinated Li cations would have on the speciation of **1** in solution, we added 12-crown-4 to a solution of the uranium alkoxide in THF (Figure S8). The emission spectrum of **1** did not show any significant change in the presence of the crown ether. Moreover, calculated spectra on model complexes with and without explicit THF and Li ions resulted in consistent spectra supporting that this dynamic exchange would only contribute to spectral broadening (Figures S9 and S10).

An alternative justification for the observed biexponential decay is needed. We return to calculations to gain insight into the origin of the observed electronic transitions in the emission spectrum. The ^3^P state populated in the absorption process involves strong spin–orbit coupling with significant triplet contributions. One contribution to the biexponential PL decay arises from the spin-forbidden nature of the initial transition, which then becomes only partially allowed via spin–orbit coupling. This has been shown to lead to long lifetimes in lanthanide complexes.^73^ We note that while the specific transitions involved in the emission of **1** are different compared to the halide complexes studied by Hashem et al.,^45^ we acknowledge that their proposal describing that long lifetimes arise from the population of two excited states that are sufficiently close in energy, could also be operative in complex **1**. The observed deviation from single exponential behavior that we observe would then likely be due to a mixture of excited state population, which is facilitated by spin–orbit coupling. Indeed, there are SO-XMS-CASPT2 states close in energy to the ^3^P state from which we compute the emission spectrum (Figure 4 (top), teal transitions). For example, a SO-state at 492 nm consists of 46% triplet and 39% singlet character. While this state has a low oscillator strength for the DFT-optimized geometry, the extent to which this would change as the molecule vibrates remains unknown.

### Low Temperature Photophysical Characterization of [Li(THF)]_2_[U(O^*t*^Bu)_6_]

**Figure 5:**
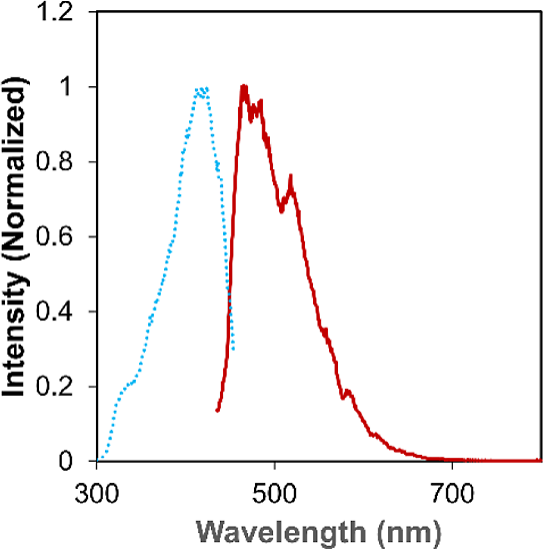
Emission (solid red line) and excitation (dashed blue line) spectra of **1** recorded in 2-MeTHF at 77 K.

We next decided to measure the photophysical properties of the alkoxide complex at low temperatures to determine if we could resolve the broadness of the emission bands in the experimental and calculated spectra. The photoluminescence of **1** measured at 77 K in a 2-methyltetrahydrofuran (2-MeTHF) glass shows partial resolution of the bands in the emission spectrum that likely correlate to individual emissive transitions. Alternatively, it is reasonable to suggest these bands may correspond to Franck–Condon progressions resulting from U–O vibronic transitions, though the resolution is not nearly to the same degree as that observed for uranyl(VI) complexes at 77 K (Figure 5). In addition, upon comparison of the low temperature data to the emission spectrum of **1** in 2-MeTHF at room temperature, a slight hypsochromic shift is observed (Figure S11). The origins of this shift are attributed to a decrease in the solvent reorganization energy at 77K. Though our calculations predict **1** to be similar to a free U^IV^ ion, with minimal contributions from the anionic tertbutoxide ligands, our experimental data suggests otherwise. The appearance of sharp peaks in the emission spectrum recorded at 77 K points to a degree of electronic coupling between the tertbutoxide ligands and metal center to produce the vibronic transitions. Therefore, there is some covalency between the aforementioned uranium–oxygen bonds, though not enough for charge transfer mechanisms to dominate the electronic transitions.

**Figure 6:**
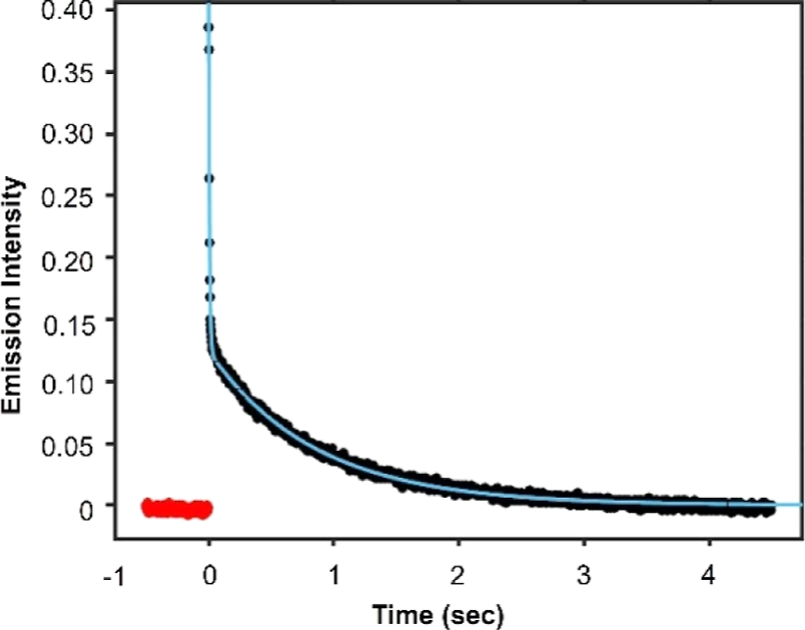
Time profile for **1** collected at 77 K in 2-MeTHF. The black trace is the experimental data and the blue trace is the fit of the curve. The red trace is time points collected before initial lamp flash.

The lifetime of complex **1** measured at 77 K in 2-MeTHF can be fit to a biexponential decay, revealing an extraordinarily long-lived excited state (τ_1_ = 0.85 s) and a second excited state that is shorter (τ_2_ = 7.5 ms) (Figure 6). These values are unprecedented in reports of the luminescence of uranium complexes, where charge-transfer mechanisms typically dominate and lead to shorter lifetimes. Indeed, even the excited state of the [UO_2_]^2+^ ion [O_yl_ →U(5f)], which is regarded for its long radiative lifetimes in frozen solution, decays on the order of hundreds of microseconds, substantially shorter than that of **1**. Lifetimes on a similar order of magnitude to **1** have been reported for lanthanide complexes and are comparable to what is observed for **1** at low temperatures. For example, Doerrer and co-workers have described the luminescence of two europium complexes with perfluorinated alkoxide ligands, [K][Eu(OC_4_F_9_)_4_] and [K(THF)_2_][Eu(pin^F^)_2_(THF)_2_], that exhibit f–f excited state lifetimes of 1.230 and 0.935 ms, respectively, due to the forbidden nature of the transitions.^73^

Given the long-lived nature of the excited state for **1,** it is reasonable to hypothesize that a uranyl impurity may be the source of the unique lifetime. To test this hypothesis, we exposed a solution of **1** in THF to air, as oxidation of **1** would produce a uranyl complex. Doing so resulted in a distinct color change of the solution from teal to yellow. This observation was probed using electronic absorption spectroscopy. The UV–vis spectrum obtained highlights the disappearance of intraconfigurational f–f transitions in the visible region, and the formation of broad and intense absorption with shoulders appearing at 430 and 515 nm (Figure S12). These observations are consistent with oxidation of the uranium metal center. Subsequent analysis of the photoluminescence spectrum of this solution produced an emission maximum at 545 nm, notably red shifted by 45 nm from that measure for **1**. Moreover, time-resolved luminescence measurements did not produce a signal on the microsecond timescale, suggesting that the decay mechanism is fast and that the uranyl ion is not the source of emission in **1**. However, to be certain that the uranyl ion would display different photophysical properties compared to **1**, the independent synthesis and photophysical characterization of a uranyl tertbutoxide complex was performed (Figure S13). Excitation of [UO_2_(O^*t*^Bu)_2_] at 450 nm at room temperature in THF resulted in photoluminescence centered at 575 nm, red shifted 75 nm from the emission maximum for **1**. In addition, the lifetime of the uranyl tertbutoxide complex measured at 77 K in 2-MeTHF did not produce signal on the microsecond time scale, further proving that a uranyl-based impurity in solution is not the source of the long decay measured for **1**, but rather a unique feature of the uranium(IV) alkoxide complex itself.

**Figure 7:**
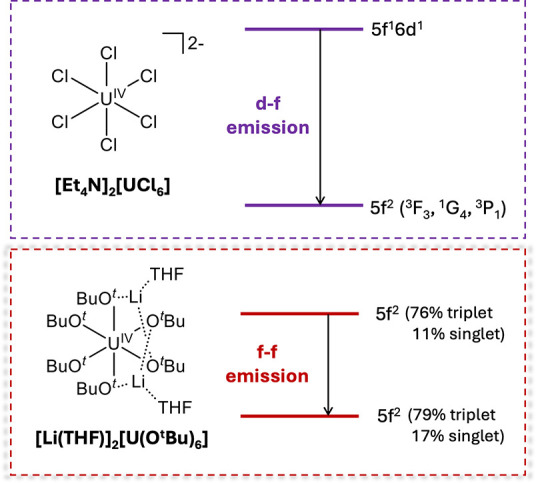
Comparison of the emission pathways for [UCl_6_]^2–^ (charge transfer and d-orbital character excited state, top) and [U(O^*t*^Bu)_6_]^2–^ (5f^2^ excited state, bottom).

Our results are consistent with the following description of the excited state dynamics of **1**: at room temperature, excitation of **1** in the f-orbital manifold results in the population of a spin–orbit state arising from energetically similar triplet and singlet spin-free excited states, and the resultant emission is significantly impacted by nonradiative transitions. This results in a relatively fast deactivation of these excited states, which is typical for actinide complexes.^71,74^ However, when nonradiative pathways are suppressed at 77 K, we observe long-lived excited state lifetimes of **1**, one of which approaches up to 1 s. We note that we cannot rule out a delayed luminescence mechanism at low temperature, whereby excited state population is sequestered in a dark state that slowly repopulates the bright excited state leading to the observed long lifetime. Indeed, our calculations indicate that some of the spin–orbit excited states close in energy to the initially excited state have low oscillator strength (vide supra). A comparison of the photophysical properties of **1** compared to other actinide complexes is included in Table 1. In addition, a comparison of the emission pathways for **1** compared to the [UCl_6_]^2–^ anion are included in Figure 7, to highlight key differences in the photophysical properties of the two tetravalent uranium complexes.

**Table 1:** Comparison of Spectral Profiles and Luminescence Lifetimes for Uranium Complexes

	[U(O^*t*^Bu)]_6_^2–^	[UO_2_(O^*t*^Bu)_2_]	[UCl_6_]^2–^	U^4+^ (aq)
transition	f–f	LMCT	LMCT	f–f
λ_abs_ (nm)	425	450	303	245
λ_em_ (nm)	500	575	364, 424, 510	290–525
Stokes’ Shift **(nm)**	75	125	61	45
τ	^a^6.7 ns	<100 μs	2–10 ns	149 ns
	^b^0.85 s			
Temperature	^a^298 K	77 K	298 K	77 K
	^b^77 K			
REF	*this work*	*this work*	(45)	(46)

### Photophysical Characterization of [Li(Et_2_O)][U^V^(O^*t*^Bu)_6_] and [U^VI^(O^t^Bu)_6_]

In their original report, Hayton and co-workers also isolated homoleptic U(V) (5f^1^) and U(VI) (5f^0^) alkoxide complexes, [Li(Et_2_O)][U^V^(O^*t*^Bu)_6_] and [U^VI^(O^*t*^Bu)_6_], to probe changes in the electronic structure of the complexes upon changes in f-orbital occupancy as the uranium center was oxidized. We saw these complexes as a convenient entry point into determining the effect that population of the 5f orbitals has on the subsequent photophysical properties of uranium alkoxides. We first opted to collect the absorption spectra of [Li(Et_2_O)][U^V^(O^*t*^Bu)_6_] and [U^VI^(O^*t*^Bu)_6_] before analyzing their photoluminescence (Figure S14). The electronic absorption spectrum of a solution of [U^V^(O^*t*^Bu)_6_]^1–^ in THF exhibits a single, broad featureless band at 230 nm (ε = 1600 M^–1^ cm^–1^), with a shoulder at 322 nm (ε = 331 M^–1^ cm^–1^). Additionally, when the solution is concentrated there are no observable intraconfigurational f–f bands from 400 to 800 nm. Analysis of the near-infrared region of [U^V^(O^*t*^Bu)_6_]^1–^ reveals relatively broad f–f transitions, consistent with the presence of a U(V) metal center. Moreover, the electronic absorption spectrum of [U^VI^(O^*t*^Bu)_6_] in THF displays two structured bands centered at 228 nm (ε = 7317 M^–1^ cm^–1^) and 290 nm (ε = 6648 M^–1^ cm^–1^), with a featureless shoulder at 358 nm (ε = 3795 M^–1^ cm^–1^) and a low intensity, broad band at 560 nm (ε = 516 M^–1^ cm^–1^), all most likely charge transfer transitions based on the high values of the molar extinction coefficients.

Analysis of [Li(Et_2_O)][U^V^(O^*t*^Bu)_6_] and [U^VI^(O^*t*^Bu)_6_] in THF under ultraviolet irradiation reveals no appreciable emission in the visible region from either complex (Figure S15). In addition, excitation with higher energy (250–350 nm) and lower energy light (380–500 nm) produced the same result, suggesting that there is not sufficient population of the excited state triplet to observe emission from the complexes. We hypothesize that the lack of competing absorption from the tert-butoxide moieties in the visible region of **1** is a crucial factor contributing to its photophysical properties. As **1** is oxidized and the symmetry of the complex is increased, the enhanced electronic communication between uranium and the tert-butoxide ligands promotes charge transfer excited states that provide additional pathways for internal conversion, resulting in rapid nonradiative decay to the ground state and quenching of luminescence.

## Conclusions

Here, we present the photophysical characterization of a homoleptic uranium(IV) tertbutoxide complex. Excitation of [Li(THF)]_2_[U^IV^(O^*t*^Bu)_6_] leads to the first example of photoluminescence from an actinide complex originating from an f–f excitation in nonaqueous solution, supported by SO-XMS-CASPT2 calculations. These calculations show strong spin–orbit coupling between the excited triplet and singlet states for the 5f-orbital manifold in **1**. We hypothesize that it is the nature of the excited state that results in an exceptionally high quantum yield of 10% for complex **1**, as well as an unprecedented, excited state lifetime of 0.85 s at 77 K. The high energy absorptions of the LMCT events between the alkoxide ligands and uranium(IV) metal center in **1** appears to be important to the photophysical properties of this compound. Shifting of these transitions to higher energy enables f–f absorption events at ∼400 nm, turning on the emission of the low-valent uranium complex. To affirm this hypothesis, we also studied previously reported homoleptic uranium(V) and uranium(VI) tertbutoxide complexes; oxidation of **1** results in quenching of luminescence in [Li(Et_2_O)][U^V^(O*^t^*Bu)_6_] and [U^VI^(O^*t*^Bu)_6_].

Overall, this combined experimental and theoretical investigation highlights the importance that the degree of covalency in uranium-ligand bonds has on the photophysical properties of uranium complexes. By synthesizing complexes with lower symmetry, we aimed to reduce orbital mixing with the ligand and, in turn, limit opportunities for charge transfer mechanisms that have previously governed the photoluminescence of uranium complexes. This allows for metal-based electronic configurations to dominate electronic transitions, unveiling the unique properties of f-centered excited states. Moving forward, we are interested in further probing the electronic structure of homoleptic uranium(IV) complexes via modulation of the ligand framework and increasing the covalency of the bonds between the uranium center and ligands by substitution of sulfur for oxygen atoms. We aim to gain further insight into the effects of electronic structure on the emission dynamics of uranium(IV) complexes, information which is critical to understanding the nature of bonding within the actinides and their ligand sets. Summarily, we believe that with the appropriate choice of ligand, actinide-based f–f states can be unveiled and studied in further depth for fundamental information on the electronic structure of actinide-ligand complexes.

## Supporting Information Available

Furthermore, all input and output files from computational work are made available in a FigShare repository (Temporary link for review only. Published link to be added upon acceptance). https://figshare.com/s/ba92c1a1ff1925a0d6b6. Additional experimental procedures, spectroscopic and crystallographic data, and computational details (PDF)
