# Supplementary material for: Photoluminescence of a Uranium(IV) Alkoxide Complex
Source: JACS Au. 2024 Dec 12;5(1):332–42. doi: 10.1021/jacsau.4c01022 (PMC11775688; doi:10.1021/jacsau.4c01022)
Supplement: Supplementary file 1 — au4c01022_si_001.pdf [file au4c01022_si_001.pdf]

Electronic Supporting Information

Photoluminescence of a Uranium(IV) Alkoxide Complex

Leyla R. Valerio,[1] Sabyasachi Roy Chowdhury,[2,3] Rob Lewis,[1] Kathryn E. Knowles,[1]* Bess Vlaisavljevich,[2,3]* Ellen M. Matson[1]*

[1] Department of Chemistry, University of Rochester, Rochester NY 14627 USA

[2] Department of Chemistry, University of South Dakota, Vermillion, South Dakota 57069, USA

[3] Department of Chemistry, University of Iowa, Iowa City, Iowa, 52240, USA

Supporting Information Table of Contents

Table S1. (2e7o)-SA-CASSCF/XMS-CASPT2 computed 21 triplet states of **1**……………………………..……**S3**

Table S2. (2e7o)-SA-CASSCF/XMS-CASPT2 computed 28 singlet states of **1**……………….…..…………**S4**

Table S3. Spin-orbit coupled energy levels of **1**……………………..……………………………………..……**S5**

Figure S1. Jablonski diagram………………………………………………………………………………..……**S6**

Figure S2. CASSCF active natural orbitals of **1** …….………………………………………………..……………**S7**

Figure S3. Excitation monitored at varying emission wavelengths……..……………………………………**S8**

Figure S4. Quantum yield calculation…………………………………………………………………..……..**S9**

Figure S5. TCSPC of **1** at room temperature……………………………………………………..……………**S10**

Figure S6. Variable temperature ^{1}H NMR studies of **1** ………………………..……………………………**S11**

Figure S7. Solution-phase studies of **1** with excess Li cations…………………..……………………………**S12**

Figure S8. Solution-phase studies of **1** in the presence of 12-crown-4………..……………………………**S13**

Figure S9. Modelling with and without THF…………………………………………………………………**S14**

Figure S10. Modelling with and without Li……………………..……………………………………………**S15**

Figure S11. Emission of **1** at 298K vs 77K……………………………………………..…………………..**S16**

Figure S12. UV-Vis and emission spectra of **1** after exposure to air…………………..…………………..**S17**

Figure S13. UV-Vis and emission spectra of [UO_2(O^{t}Bu)$_2$] ……………………………………..……..……**S18**

Figure S14. Electronic absorption of [Li(THF)][U(O^{t}Bu)$_6$] and [U(O^{t}Bu)$_6$] under UV irradiation……..……**S19**

Figure S15. [Li(THF)][U(O^{t}Bu)$_6$] and [U(O^{t}Bu)$_6$] under UV irradiation……………………….………………**S20**

Table S1. (2e7o)-SA-CASSCF/XMS-CASPT2 computed 21 triplet states of complex 1.

Index	Energy (a.u)	Energy (cm^{-1})
1	-29821.18639870	0
2	-29821.18375048	581.2
3	-29821.18276376	797.7
4	-29821.18110792	1161.2
5	-29821.17768216	1913.2
6	-29821.17756317	1939.2
7	-29821.17638883	2196.9
8	-29821.17243205	3065.3
9	-29821.17209768	3138.7
10	-29821.17103693	3371.5
11	-29821.16588453	4502.3
12	-29821.16079909	5618.5
13	-29821.15709272	6431.9
14	-29821.15639194	6585.7
15	-29821.15429097	7046.8
16	-29821.15321841	7282.2
17	-29821.15085156	7801.7
18	-29821.14787319	8455.4
19	-29821.10856027	17083.6
20	-29821.10613979	17614.8
21	-29821.10514729	17832.7

Table S2. (2e7o)-SA-CASSCF/XMS-CASPT2 computed 28 singlet states of complex 1.

Index	Energy (a.u)	Energy (cm^{-1})
1	-29821.17782106	1882.6
2	-29821.16863591	3898.5
3	-29821.16556231	4573.1
4	-29821.16408794	4896.7
5	-29821.16302809	5129.3
6	-29821.16023891	5741.4
7	-29821.15465875	6966.1
8	-29821.15186685	7578.9
9	-29821.14788354	8453.1
10	-29821.14146722	9861.3
11	-29821.13869106	10470.6
12	-29821.13734092	10766.9
13	-29821.12912453	12570.2
14	-29821.12169695	14200.4
15	-29821.11720856	15185.5
16	-29821.11475076	15724.9
17	-29821.11465413	15746.1
18	-29821.11287973	16135.6
19	-29821.11177873	16377.2
20	-29821.10904614	16976.9
21	-29821.10859865	17075.2
22	-29821.10223258	18472.4
23	-29821.10170707	18587.7
24	-29821.09667522	19692.1
25	-29821.09559551	19928.9
26	-29821.09485406	20091.8
27	-29821.09449583	20170.4
28	-29821.03195867	33895.7

Table S3. The spin-orbit coupled energy levels of complex 1 together with the oscillator strengths from the ground spin-orbit state.

Index	Energy (cm^{-1})	Energy (nm)	Oscillator Strength
1	0.0	--	--
2	1175.5		1.45446866E-06
3	1367.6	8507.02	6.36968870E-08
4	1435.5	7311.54	1.30736760E-06
5	1468.4	6966.21	2.41600363E-06
6	1662.4	6810.13	7.47713066E-08
7	3133.9	6015.40	7.04933711E-08
8	3170.8	3190.91	1.59594242E-06
9	3547.4	3153.78	5.25645262E-06
10	4986.8	2818.97	1.81802226E-07
11	5431.8	2005.29	1.44526516E-05
12	6135.3	1840.98	1.65533950E-05
13	6258.9	1629.91	1.26383336E-05
14	6366.2	1597.72	4.12367344E-05
15	7281.3	1570.80	2.32913105E-06
16	7328.3	1373.38	1.83810623E-07
17	7747.2	1364.57	1.40612067E-06
18	8007.6	1290.79	2.40235638E-05
19	8162.5	1248.81	1.00638069E-05
20	8228.0	1225.11	5.10484330E-07
21	8412.1	1215.36	4.99236295E-06
22	8485.7	1188.76	2.46245280E-06
23	8520.7	1178.45	2.13567683E-05
24	8898.4	1173.61	1.70779325E-07
25	9119.9	1123.80	2.50105171E-07
26	9289.5	1096.50	1.25801925E-05
27	10427.6	1076.48	7.14881576E-06
28	10451.9	958.99	1.73443587E-05
29	10542.6	956.76	1.50057607E-07
30	10754.8	948.53	1.86370795E-06
31	10791.8	929.81	6.84004727E-07
32	10974.0	926.62	3.55941535E-09
33	11205.6	911.24	1.23028739E-05
34	11281.5	892.41	1.29944500E-05
35	11581.3	886.41	1.16064240E-06
36	11678.1	863.46	3.01843612E-06
37	11894.7	856.30	7.43909583E-06
38	12042.4	840.71	1.45225699E-07
39	12519.1	830.40	2.84611850E-06
40	12832.1	798.78	2.99697023E-07
41	12884.6	779.30	1.19623125E-07
42	12916.2	776.12	2.13517471E-06
43	13180.8	774.22	1.28218559E-06
44	13201.6	758.68	3.47978860E-06
45	13278.8	757.48	1.39722329E-07
46	13348.2	753.07	4.47978138E-07
47	13903.8	749.16	9.73360564E-07
48	14512.2	719.22	7.34759861E-08
49	14595.0	689.08	4.76998286E-06
50	15111.1	685.17	9.34828178E-08
51	15399.5	661.77	1.27014362E-05

52	15682.3	649.37	1.62441751E-05
53	15818.3	637.66	1.17135250E-07
54	16270.7	632.18	9.60118838E-07
55	16419.3	614.60	8.45086894E-07
56	17006.0	609.04	3.59913953E-05
57	17416.7	588.03	9.88482273E-08
58	17588.5	574.16	2.50328246E-05
59	17731.8	568.55	1.88527287E-05
60	17913.3	563.96	1.59223333E-07
61	17994.3	558.24	4.11511072E-06
62	18188.6	555.73	5.17438225E-06
63	18211.4	549.79	1.13842192E-05
64	19358.1	549.11	5.44673201E-06
65	19529.3	516.58	9.37768111E-07
66	19720.9	512.05	3.07504764E-06
67	19867.7	507.08	9.13615060E-07
68	20299.6	503.33	1.24613163E-05
69	20576.9	492.62	8.78791542E-05
70	23061.6	485.98	5.56686907E-06
71	23118.9	433.62	2.38897960E-05
72	23255.7	432.54	5.84578519E-06
73	23315.6	430.00	2.07872307E-05
74	23421.6	428.90	1.09546519E-05
75	23456.8	426.96	7.14360012E-06
76	23731.9	426.32	5.45519224E-06
77	24094.7	421.37	2.19297524E-06
78	24563.3	415.03	7.01013545E-07
79	24657.3	407.11	2.46555424E-06
80	24989.6	405.56	1.34553348E-05
81	25479.0	400.17	1.17106451E-06
82	26051.0	392.48	1.97210699E-05
83	26110.7	383.86	1.53242532E-05
84	26372.5	382.98	8.93534895E-07
85	26703.5	379.18	1.24237573E-05
86	27280.8	374.48	1.45013419E-05
87	27939.4	366.56	2.71663455E-07
88	28039.1	357.92	2.60540823E-06
89	29002.7	356.64	2.67976160E-06
90	29238.3	344.79	2.86591557E-06
91	42794.3	342.02	2.76842703E-05
		233.68	

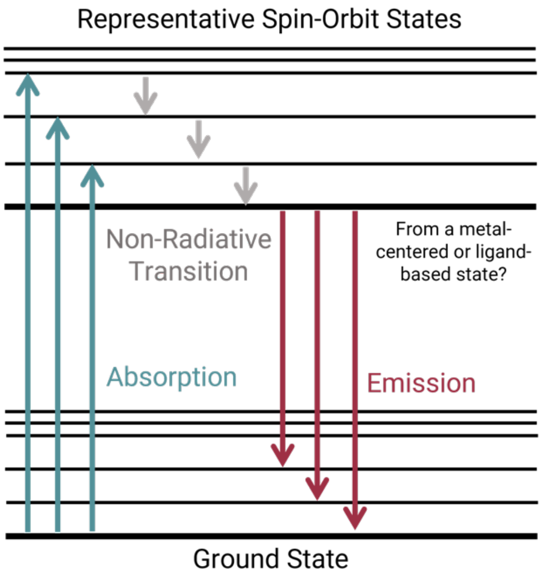

Figure S1. Jablonski diagram of the spin-orbit states involved in emission processes. It is the nature of the specific state involved in the transition that drives the nature of the mechanism.

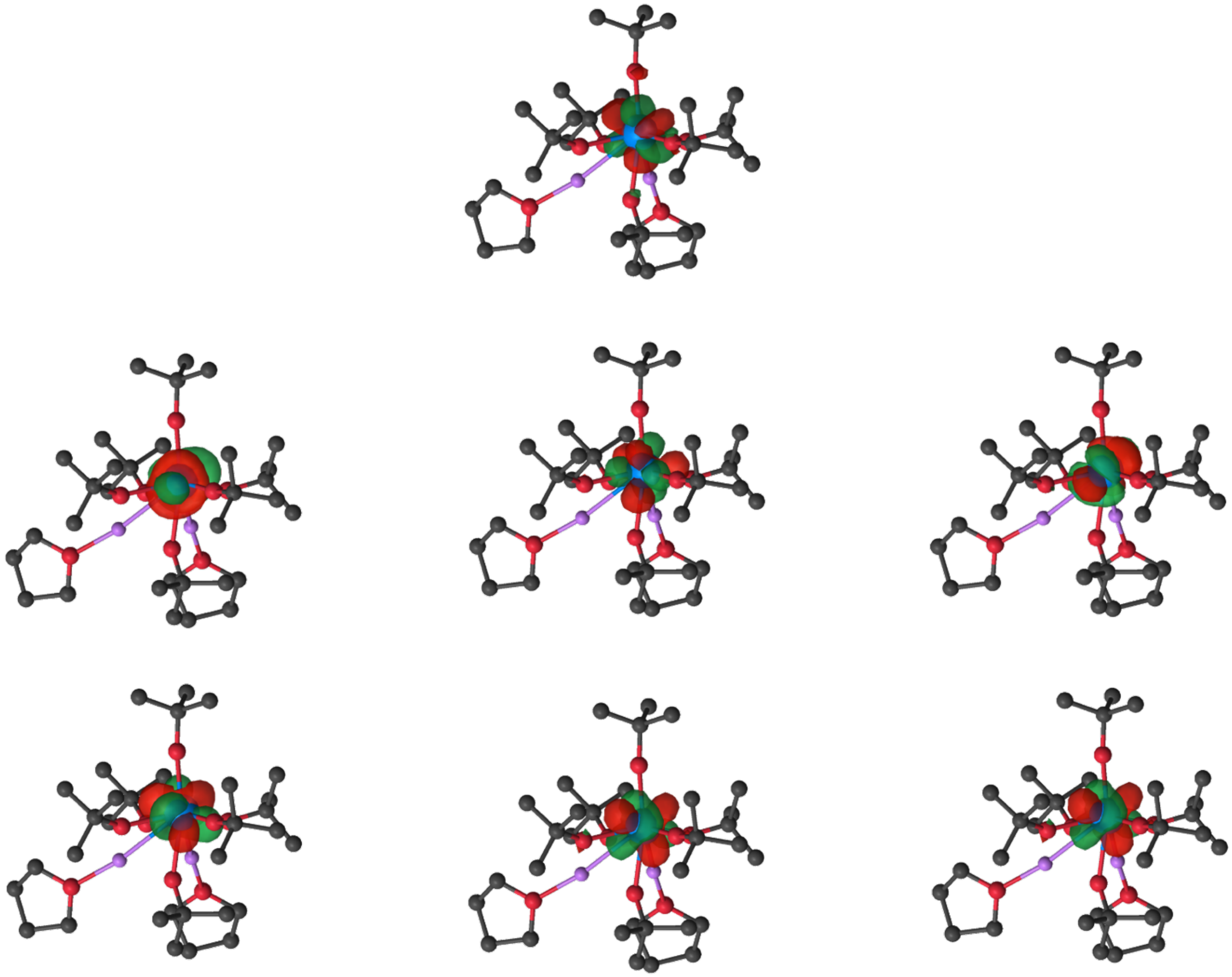

Figure S2. The CASSCF active natural orbitals of the complex **1** from the (2e, 7o) active space. An isosurface value of 0.04 a.u. was used.

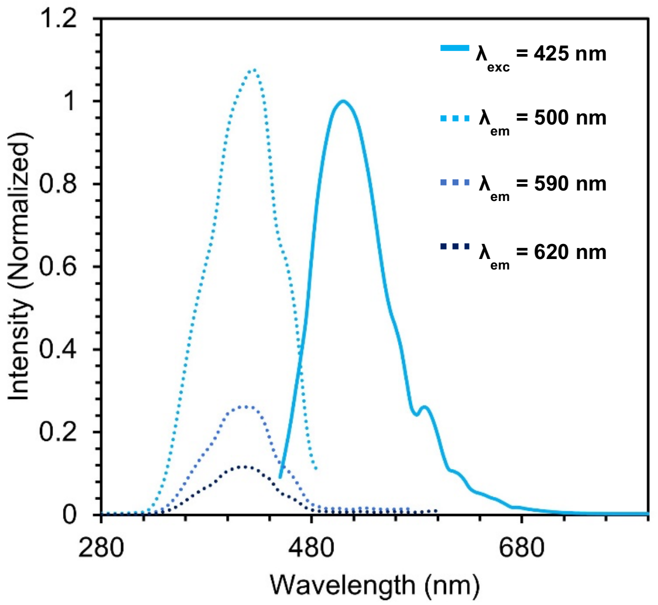

Figure S3. Excitation spectra of **1** monitored at 500 nm, 590 nm, and 620 nm emission, showing that all peaks in emission spectrum correspond to the same excited state.

Quantum Yield Determination

The quantum yield of 1 was determined via comparative method. Coumarin-540 in ethyl acetate (Φ = 0.88) was used as a standard. The quantum yield was calculated using the following equation:

The subscripts *X* and *Y* stand for the sample and reference, respectively. Φ is the photoluminescence

$$\Phi_X = \Phi_Y * \left(\frac{Absorbance_Y}{Absorbance_X}\right)\left(\frac{\Sigma\, Emission_X}{\Sigma\, Emission_Y}\right)\left(\frac{\eta_X}{\eta_Y}\right)^2$$

quantum yield. η is the refractive index of the solvent.

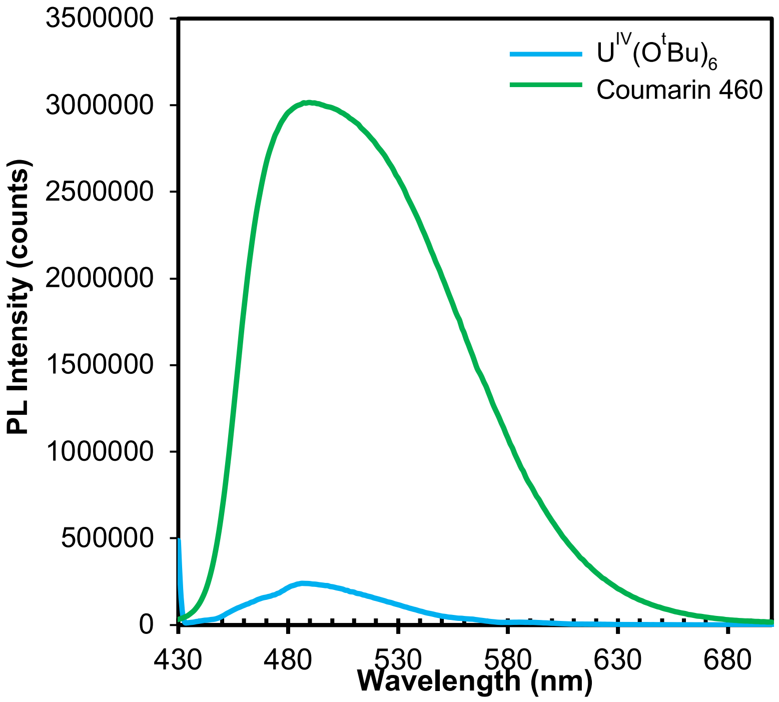

Figure S4. Emission of Coumarin-540 in ethyl acetate plotted with **1** for determination of quantum yield via the comparative method.

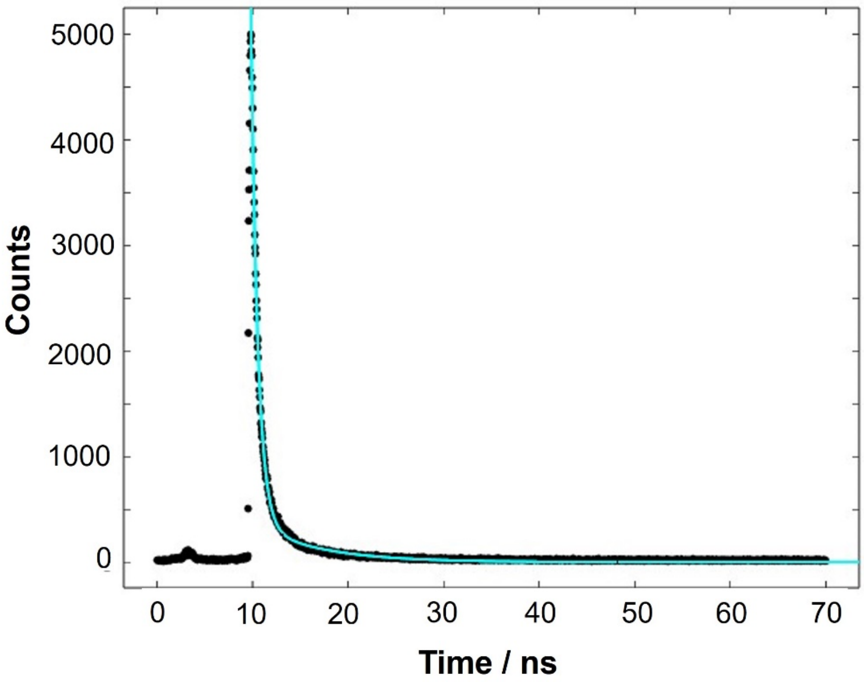

Figure S5. Time profile for **1** collected at room temperature in THF. The black trace is the experimental data and the blue trace is the fit of the curve.

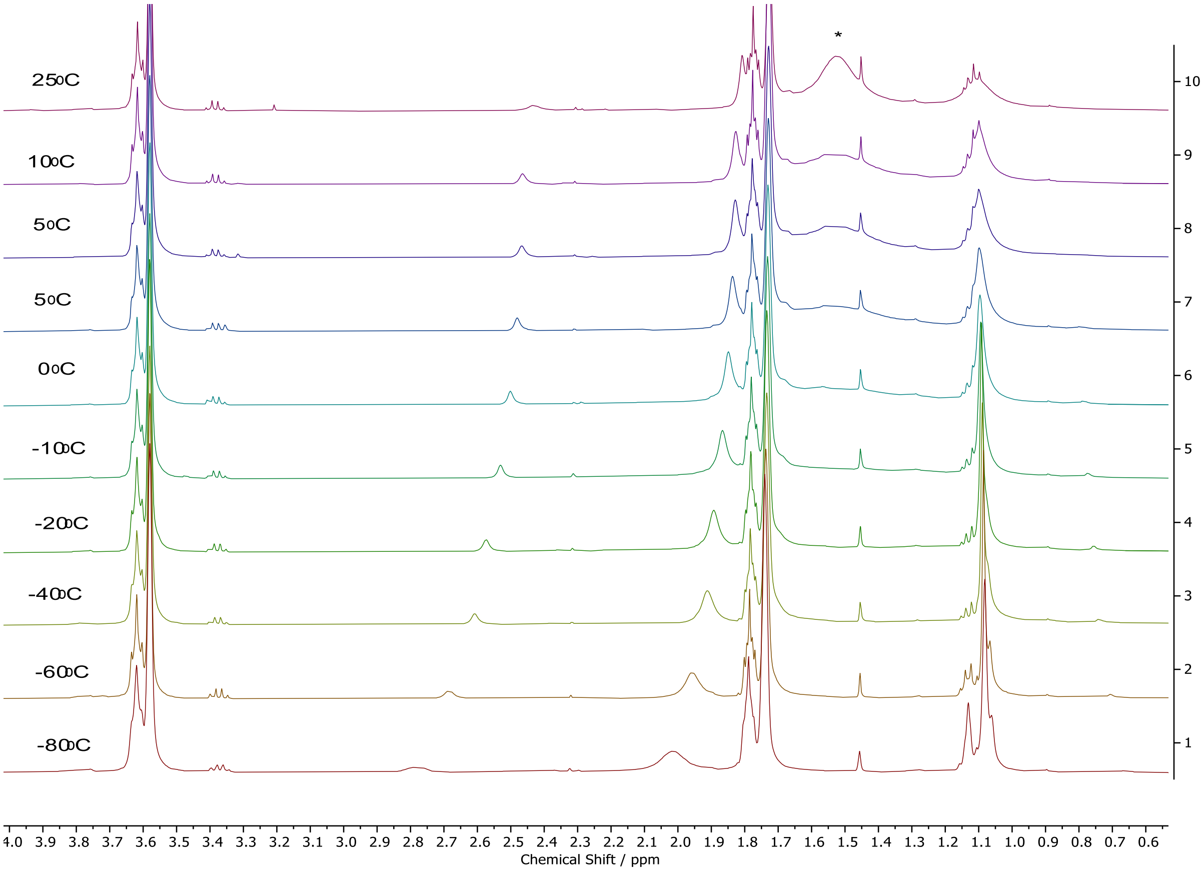

Figure S6. Variable temperature ^{1}H NMR of **1** in THF-d_8.

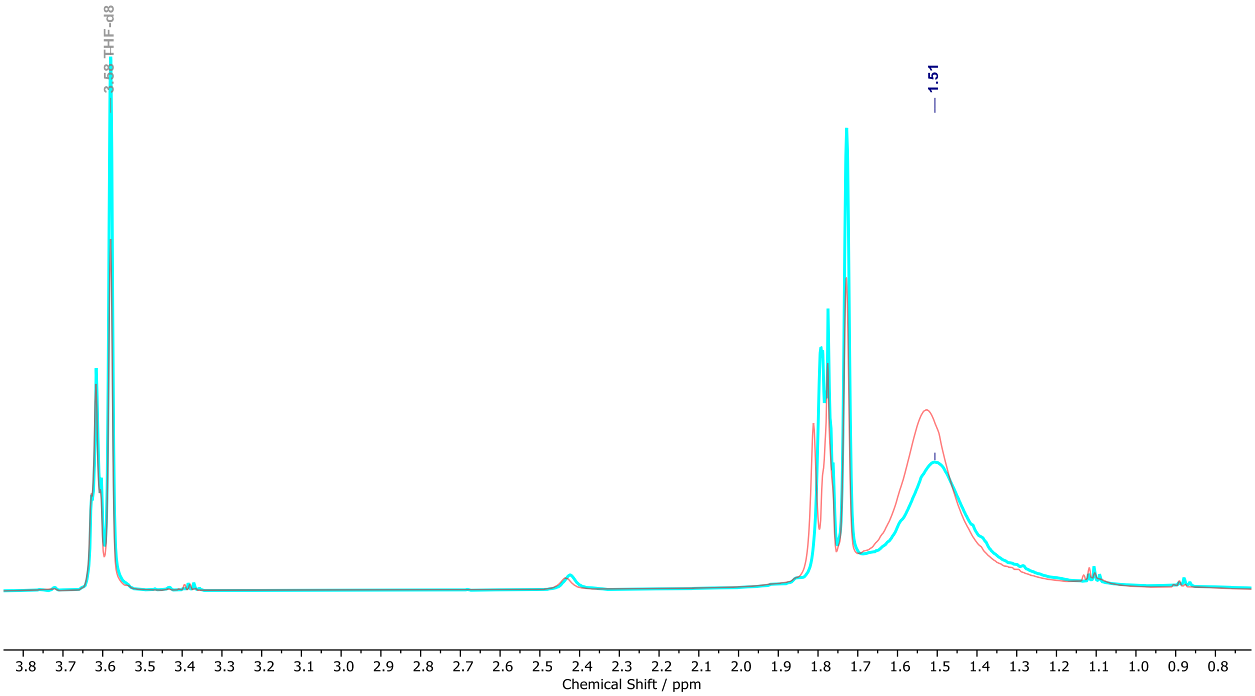

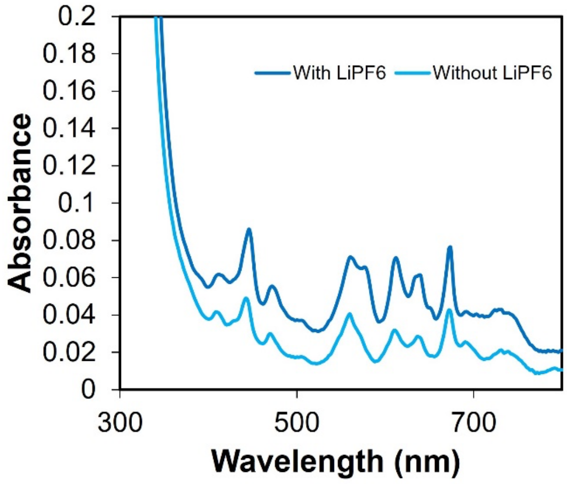

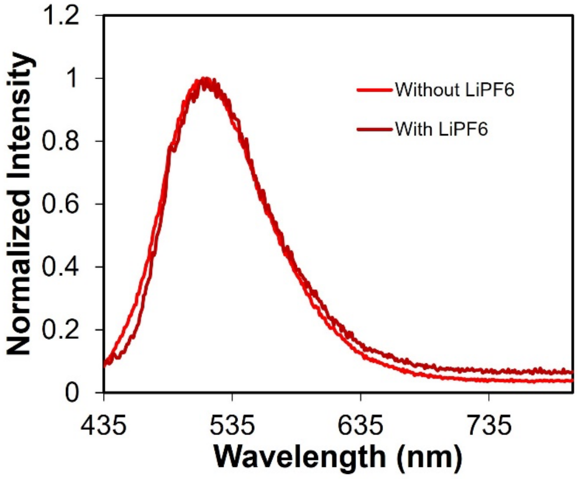

Figure S7. (top) ^{1}H NMR of **1** in THF-d_8 with an excess of $LiPF_6$ added in solution (teal). (bottom) Electronic absorption and emission spectra of **1** in THF with an excess of $LiPF_6$ added in solution.

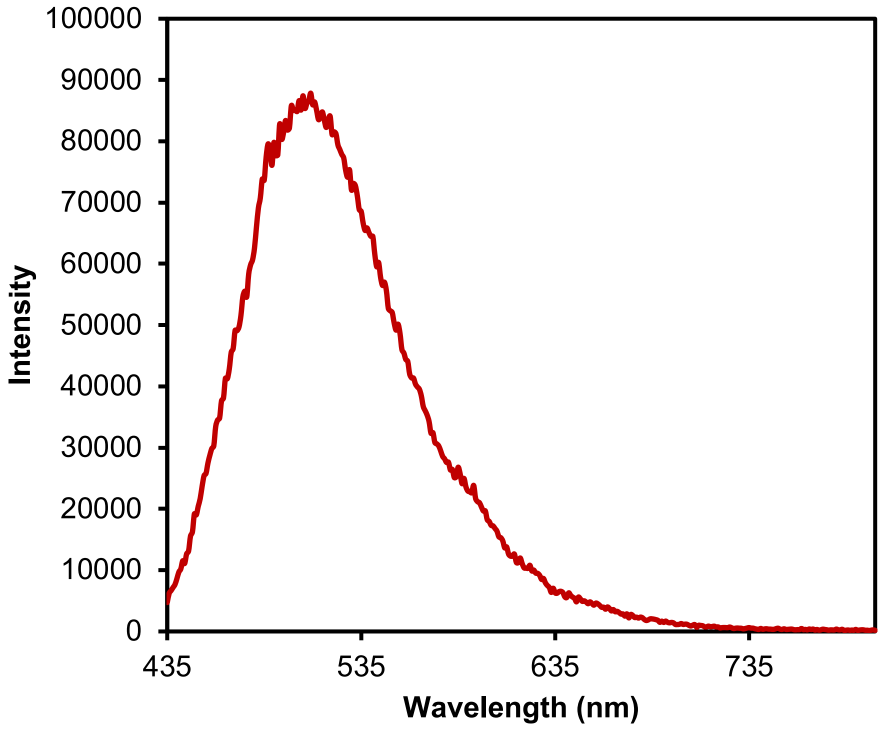

Figure S8. Emission spectrum of **1** in the presence of 12-crown-4.

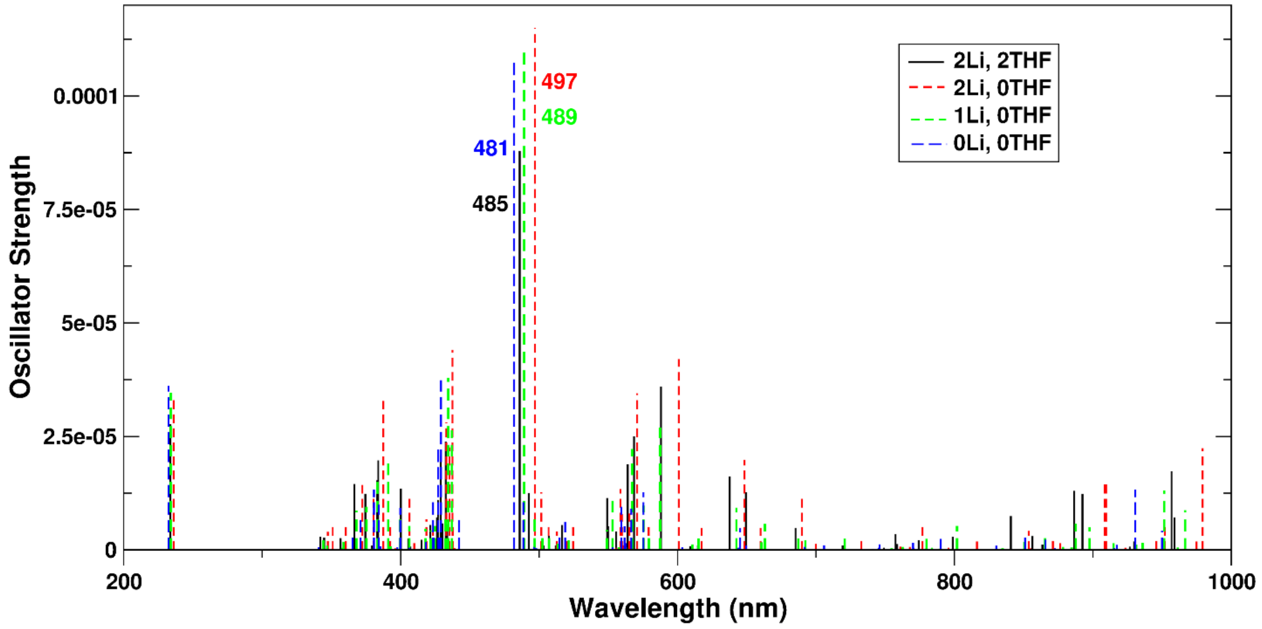

Figure S9. The SO-XMS-CASPT2 computed absorption spectra (line spectra) of **1,** with and without explicit Li ions and THF. The transition with the largest intensity is labeled.

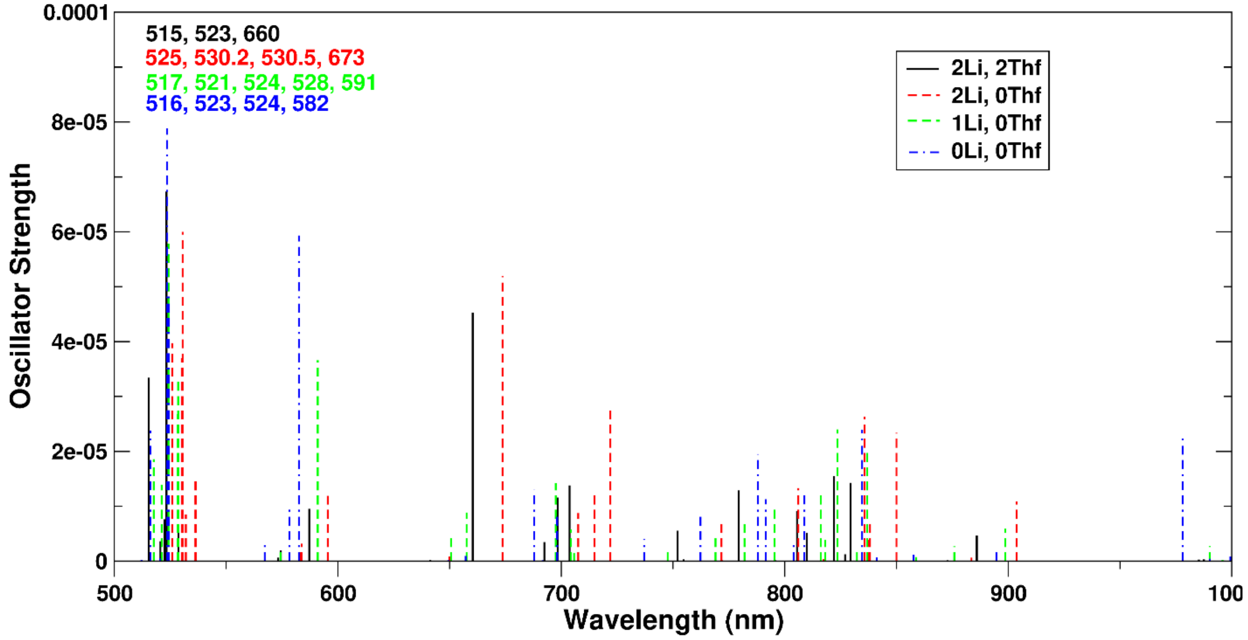

Figure S10. The SO-XMS-CASPT2 computed emission spectra (line spectra) of **1,** with and without explicit Li ions and THF. The transitions with the largest intensities are labeled.

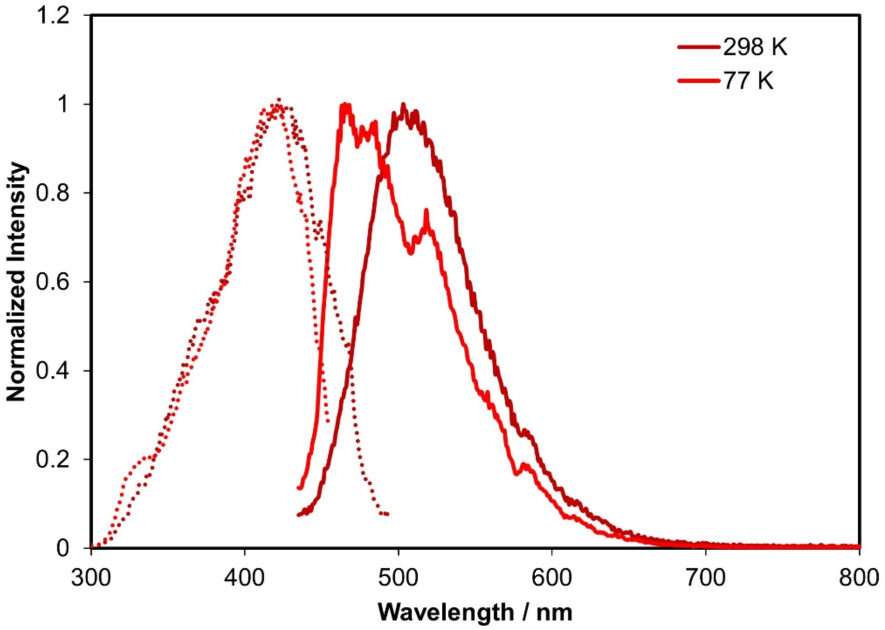

Figure S11. Excitation and emission spectra of **1** in 2-MeTHF at 298 K and 77 K.

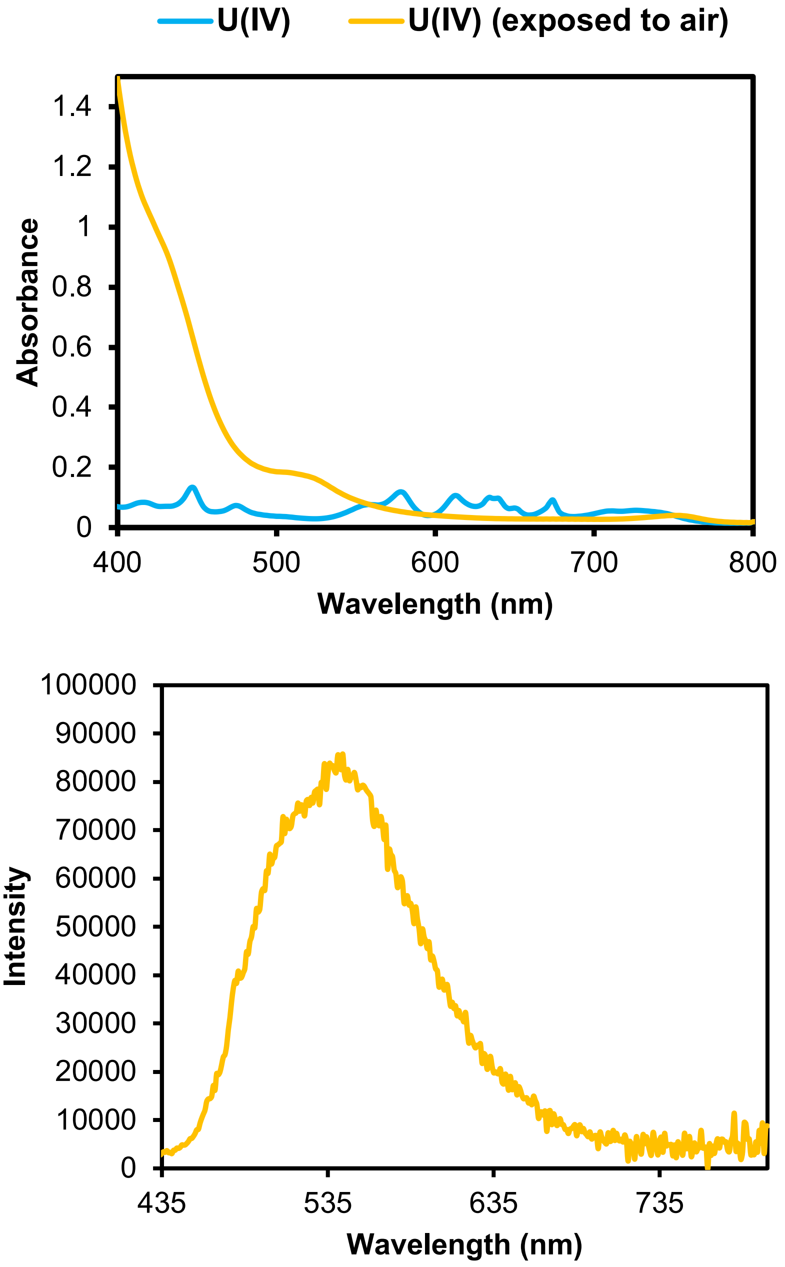

Figure S12. (top) Electronic absorption spectrum of **1** (blue trace) recorded in anhydrous THF at room temperature before exposure to air. The yellow trace is the electronic absorption spectrum of the solution recorded after exposure to air, indicating oxidation has occurred. (bottom) Emission spectrum of the solution exposed to air in anhydrous THF (λ_{exc} = 425 nm).

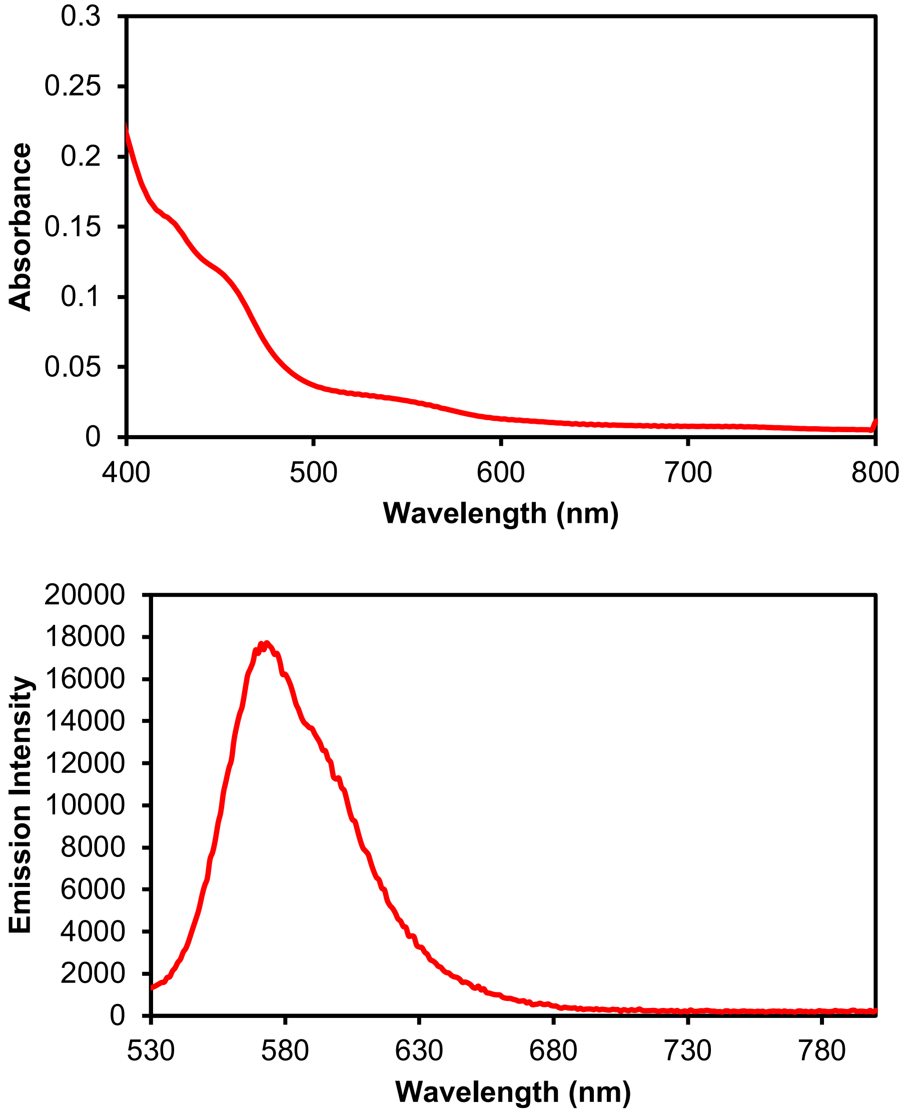

Figure S13. (top) Electronic absorption spectrum of [$UO_2(O^tBu)_2$] recorded in anhydrous THF at room temperature. (bottom) Emission spectrum of [$UO_2(O^tBu)_2$] recorded in anhydrous THF at room temperature (λ_{exc} = 450 nm).

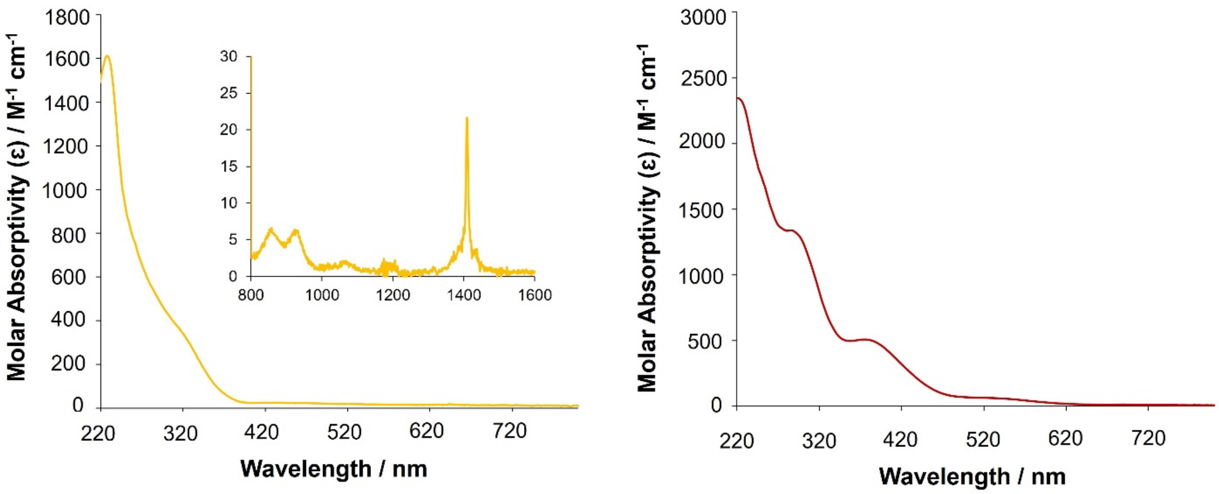

Figure S14. Electronic absorption spectra of [Li(THF)][U(O^{t}Bu)$_6$] and [U(O^{t}Bu)$_6$] in THF.

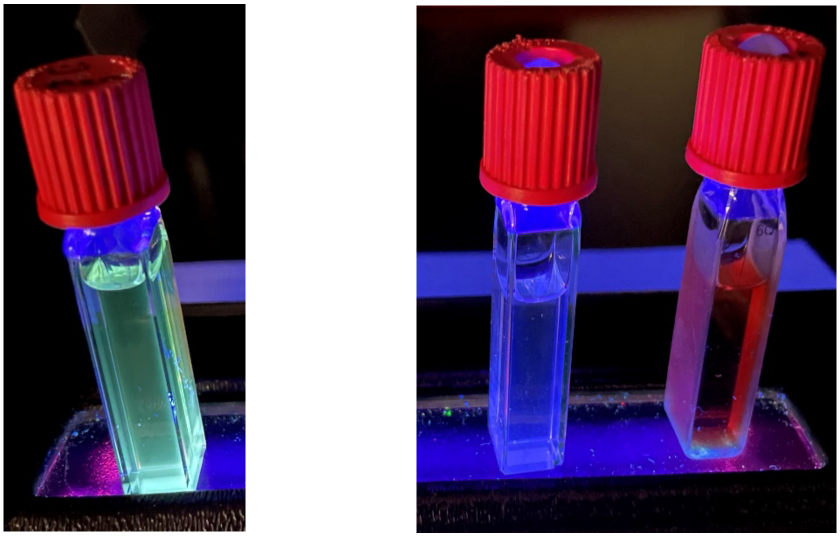

Figure S15. (left) **1** in THF irradiated under UV light. (right) [Li(THF)][U(O^{t}Bu)$_6$] and [U(O^{t}Bu)$_6$] irradiated under UV light.
